# Medical visits and mortality among dementia patients during the COVID-19 pandemic compared to rates predicted from 2019

**DOI:** 10.1186/s12877-024-05298-2

**Published:** 2024-09-02

**Authors:** Kaushik Ghosh, Susan T. Stewart, Trivellore Raghunathan, David M. Cutler

**Affiliations:** 1 National Bureau of Economic Research, Cambridge, MA 02138 USA; 2 Institute for Social Research and Department of Biostatistics, University of Michigan, Ann Arbor, MI 48106 USA; 3 Department of Economics, Harvard University, Cambridge, MA 02138 USA

**Keywords:** Health care use, Health care costs, Dementia, Alzheimer’s, COVID-19, Mortality, Deaths, Primary care, Inpatient, Emergency care, Long-term care, Doctor visits, Telehealth, Medicare

## Abstract

**Background:**

During the COVID-19 pandemic, patients with Alzheimer’s disease and related dementias (ADRD) were especially vulnerable, and modes of medical care delivery shifted rapidly. This study assessed the impact of the pandemic on care for people with ADRD, examining the use of primary, emergency, and long-term care, as well as deaths due to COVID and to other causes.

**Methods:**

Among 4.2 million beneficiaries aged 66 and older with ADRD in traditional Medicare, monthly deaths and claims for routine care (doctors’ office and telehealth visits), inpatient/emergency department (ED) visits, and long-term care facility use from March or June 2020 through December 2022 are compared to monthly rates predicted from January–December 2019 using OLS and logistic/negative binomial regression. Correlation analyses examine the association between excess deaths — due to COVID and non-COVID causes — and changes in care use in the beneficiary’s state of residence.

**Results:**

Increased telehealth visits more than offset reduced office visits, with primary care visits increasing overall (by 9 percent from June 2020 onward relative to the predicted rate from 2019, *p* < .001). Emergency/inpatient visits declined (by 9 percent, *p* < .001) and long-term care facility use declined, remaining 14% below the 2019 trend from June 2020 onward (*p* < .001). Both COVID and non-COVID deaths rose, with 231,000 excess deaths (16% above the prediction from 2019), over 80 percent of which were attributable to COVID. Excess deaths were higher among women, non-White patients, those in rural and isolated zip codes, and those with higher social deprivation index scores. States with the largest increases in primary care visits had the lowest excess deaths (correlation -0.49).

**Conclusions:**

Older adults with ADRD had substantial deaths above pre-pandemic projections during the COVID-19 pandemic, 80 percent of which were attributed to COVID-19. Routine care increased overall due to a dramatic increase in telehealth visits, but this was uneven across states, and mortality rates were significantly lower in states with higher than pre-pandemic visits.

**Supplementary Information:**

The online version contains supplementary material available at 10.1186/s12877-024-05298-2.

## Background

Modes of medical care delivery shifted rapidly during the COVID-19 pandemic, with significant reductions in inpatient care [1, 2], nursing home use [3], and the widespread but geographically diverse adoption of telehealth [2, 4–10]. At the same time, the COVID-19 pandemic has been associated with significant excess mortality in the US population [11–17].

Patients with Alzheimer’s disease and related dementias (ADRD) have among the highest rates of medical care use in the country and experience one of the highest mortality rates in the population. They are also especially vulnerable given their greater likelihood of living in group settings and thus exposure to infectious diseases [17, 18]. Further, those with Alzheimer’s disease may be particularly susceptible to SARS-CoV-2 infection and mortality [19, 20].

However, little is known about overall care patterns and mortality trends for people with ADRD during the COVID pandemic. Those with mild cognitive impairment or dementia were found in one study [21] to have more significant disruptions in outpatient, emergency, and inpatient care than non-MCI/ADRD patients. Most studies examining excess deaths during the pandemic examine the whole population [12–15], just younger adults [11], or all nursing home residents [16]. Of studies focusing on those with ADRD, Gilstrap et al. [17] found that deaths among Medicare enrollees with ADRD were 26% higher in 2020 than in 2019 (vs. 12% higher for those without ADRD), but did not estimate the proportion of these deaths attributable to Covid. Lamont et al. [18] found that excess deaths in 2020 were primarily attributable to Covid. A meta-analysis including more recent data estimated that people with dementia without COVID-19 had 25% increased mortality during the pandemic [22].

This paper examines medical care use and mortality trends for patients with ADRD and differences across states and socioeconomic status. We focus on measures of paid care receipt, especially primary care, and also measures of poor outcomes – inpatient or emergency department use and COVID and non-COVID mortality. We examine trends through 2022.

## Methods

### Data/study population

In administrative data from the Centers for Medicare and Medicaid Services (CMS) for a 100% sample of beneficiaries in traditional Medicare, we identify patients with ADRD in each month who are age 66 or older and enrolled that month in fee-for-service Medicare Parts A and B, including those in residential care. Monthly health care use and mortality is tracked from January 1, 2019, through December 31, 2022.

Dementia status is recorded in the Chronic Conditions Data Warehouse (CCW), which indicates diagnosed ADRD as of the end of the previous calendar year. We update dementia status during the subsequent year using claims information and the same algorithm used in forming the CCW [23]. Appendix Table 1 provides ICD-10 codes for ADRD. Beneficiary zip codes during each calendar year are identified using the Master Beneficiary Summary File.

### Utilization and mortality measures

Monthly medical care utilization is measured by the number of claims in that month in two categories: evaluation and management visits — office visits, telehealth, and the combined number of office and telehealth visits — and care occurring as a result of poor health: emergency department utilization (ED) [24] and urgent/emergency hospital admission. Total Medicare spending in the month reflects payments made by Medicare or the beneficiary (out-of-pocket) for all care a patient receives.

Mortality is measured from Medicare administrative files. The use of long-term care services (LTC) is an indicator of whether the patient is receiving care in a long-term care facility outside a rehabilitation setting in the relevant month (i.e., not a Medicare-covered skilled nursing facility stay). Appendix Table 2 gives the list of codes used to identify each of these outcome variables.

Dummy variables are created for the 24 diagnoses recorded in the CCW [23]. Demographic information includes age, gender, and race/ethnicity, as categorized using the Research Triangle Institute race and ethnicity indicator [25]. Dual eligibility for Medicare and Medicaid is used as a proxy for being a low-income beneficiary. Social Deprivation Index (SDI) [24] scores are assigned by CMS based on ZIP code by linking to 2014–2018 American Community Survey data. The SDI score ranges from 0–100, with a higher SDI score associated with the lower socioeconomic status of the area (zip code). We group the SDI into terciles to allow for non-linear effects on utilization and outcomes. Federal Rural–Urban Commuting Area (RUCA) codes from 2010 are assigned by CMS based on census tract. Other area effects are captured through dummy variables for hospital referral regions (HRR; geographic delineations created by the Dartmouth Atlas of Health Care) based on the beneficiary’s zip code of residence [26].

### Statistical analyses

All statistical analyses are conducted on patient-month data; an individual is in the sample each month from initial ADRD diagnosis, which may have been before our sample period, until death or the end of 2022. Actual Medicare utilization and deaths in each month from March 2020–December 2022 are compared to those predicted from OLS regression models using monthly data from January–December 2019. The figures show results for every month. For summary purposes we sometimes discuss average values of observed relative to expected outcomes from June 2020–December 2022, to avoid the earliest parts of the COVID era, when there was little time to prepare for COVID-19 and diagnostic testing was limited.

OLS regression was used because non-liner models are difficult to estimate with such large samples and many fixed effects. However, using a 1% sample, results were estimated using a logit model for mortality and a negative binomial model for visits. Appendix figures 1a and b show that the results are very similar to those from linear models.

Covariates used in the prediction model include SDI terciles and dummy variables for 10-year age-gender groups; race/ethnicity (non-Hispanic Black, Hispanic, Asian/Pacific Islander, other); dual Medicare-Medicaid enrollment; urban, rural, and isolated areas based on zip codes; 24 CCW chronic conditions (omitting ADRD); month of the year, to control for seasonal variation in outcomes; hospital referral region (HRR); and residence in a skilled nursing facility (SNF) for rehabilitative care in the prior month (except in the long-term care facility regression analysis). Race/ethnicity and sex are constant over time. Comorbidities are adjusted monthly. Age and location (and thus associated SDI and Urban-Rural designation) are updated annually by CMS. The models cluster standard errors at the individual level to account for correlation. Socioeconomic differences in excess deaths are determined from the regression model, by adding up observed and expected deaths among different socioeconomic groups: age, race/ethnicity, urban/rural residence status; and socioeconomic factors (dual Medicare/Medicaid eligibility and terciles of the SDI).

For mortality, deaths are divided into COVID and non-COVID causes. A death is attributed to COVID if there was a COVID diagnosis on an inpatient, skilled nursing facility, or hospice claim in the last 14 days of life. We also used a 30-day window and found similar results.

To predict care utilization and deaths by state, we use models with fixed effects for states and individual covariates. Correlation analyses examine the association between excess deaths and changes in routine or inpatient/ED visits in the beneficiary’s state of residence, measured as the difference between actual and predicted visits over 2020, 2021, and 2022. Analyses were conducted using STATA software, version 17.0, and SAS software, version 9.4.

## Results

### Sample characteristics

Table 1 shows the characteristics of our sample. There were 4.2 million people in traditional Medicare with ADRD in 2019–2022, with an average age of 82 years. About 37% were male, and 20% were Hispanic or non-White. The prevalence of most comorbid conditions was high. For example, 68% of the population had ischemic heart disease, 36% had stroke/transient ischemic attack (TIA), and 64% had a diagnosis of depression—much higher than the 18% in the overall Medicare fee-for-service population in 2018 [27]. The prevalence of cardiovascular risk factors was also high: 51% had a past diagnosis of diabetes.

**Table 1:** Characteristics of patients age 66 and older with ADRD in traditional Medicare, 2019–2022

**Number of Skilled Nursing Facility Claims (prior month)**	**0.05**
**Age-Sex**	
**Male**	
Age 65–74	10%
Age 75–84	16%
Age 85 and over	11%
**Female**	
Age 65–74	12%
Age 75–84	24%
Age 85 and over	27%
**Race/Ethnicity**	
White Non-Hispanic	80%
Black Non-Hispanic	9%
Hispanic	6%
Asian/Pacific Islander	3%
Other races	2%
**Chronic Conditions (Ever had)**	
Atrial Fibrillation	30%
Acute Myocardial Infarction (AMI)	10%
Diabetes	51%
Glaucoma	33%
Chronic obstructive pulmonary disease (COPD)	41%
Hypertension	94%
Hyperlipidemia	91%
Depression	64%
Anemia	80%
Asthma	20%
Cataract	84%
Ischemic Heart Disease (IHD)	68%
Arthritis	80%
Osteoporosis	25%
Chronic Kidney Disease	59%
Congestive Heart Failure (CHF)	48%
Cancer: Breast	8%
Cancer: Lung	2%
Cancer: Colorectal	4%
Cancer: Prostate	7%
Cancer: Endometrial	3%
Hip Fracture	12%
Stroke/Transient Ischemic Attack (TIA)	36%
Hypothyroidism	43%
**Urbanicity**	
Urban	80%
Large Rural	10%
Small Rural	6%
Isolated	4%
**Social Deprivation Index**	
Top Tercile: Least Deprived	30%
Middle Tercile:	33%
Bottom Tercile: Most Deprived	37%
**Dually Eligible for Medicare Medicaid**	24%

Data are for patients with ADRD who are age 66 or older and enrolled that month in fee-for-service Medicare Parts A and B, in administrative data from the Centers for Medicare and Medicaid Services (CMS) for a 100% sample of beneficiaries in traditional Medicare. Observations are at the patient-month level: *N* = 114,638,189 person-month observations; 5,237,349 unique beneficiaries. Federal Rural–Urban Commuting Area (RUCA) codes from 2010 are assigned by CMS based on census tract. Social Deprivation Index (SDI) scores are assigned by CMS based on ZIP code by linking to 2014–2018 American Community Survey data. The SDI score ranges from 0–100, with a higher SDI score associated with the lower socioeconomic status of the area, and is divided into terciles

### Forming predicted outcomes

The results of prediction models estimated on monthly data from January 2019–December 2019 are in Appendix Table 3 and generally conform with expectations. The clinical conditions typically affect utilization and outcomes in the expected fashion. Older people have higher mortality even conditional on medical conditions, as do people in the highest social deprivation tercile.

### Comparison of actual and predicted outcomes

**Fig. 1:**
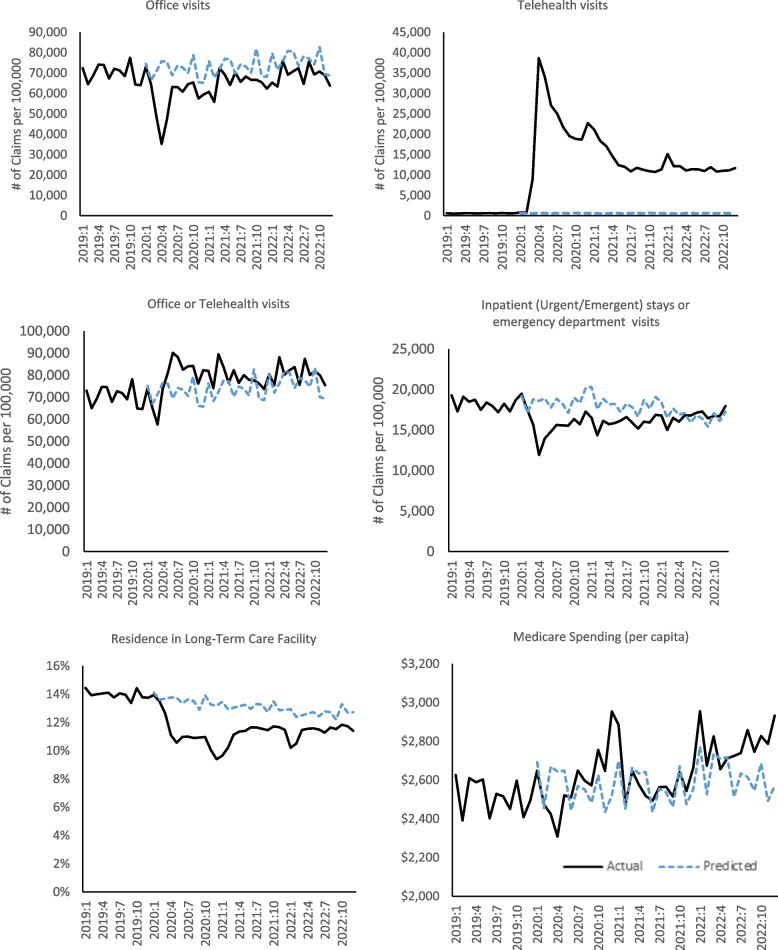
Monthly trends in actual and predicted utilization and spending for patients with ADRD during the COVID-19 pandemic, March 2020-December 2022. Notes: Dashed line is prediction from a regression model using monthly data from January–December 2019. Data are for patients with ADRD who are age 66 or older and enrolled that month in fee-for-service Medicare Parts A and B, in administrative data from the Centers for Medicare and Medicaid Services (CMS) for a 100% sample of beneficiaries in traditional Medicare. Observations are at the patient-month level: *N* = 114,638,189 person-month observations; 5,237,349 unique beneficiaries. Predicted utilization is from OLS regression models using monthly data from January–December 2019, controlling for 10-year age-gender groups, race/ethnicity (non-Hispanic Black, Hispanic, Asian/Pacific Islander, other), dual Medicare-Medicaid enrollment, Social Deprivation Index (SDI) score tercile, urban/rural/isolated areas, 24 chronic conditions, month of the year, hospital referral region (HRR), and residence in a skilled nursing facility (SNF) for rehabilitative care in the prior month (except in the long-term care facility regression analysis)

Trends in actual and predicted monthly office visits, telehealth visits, and inpatient/ emergency department visits for the population with ADRD are shown in Fig. 1. The first panel shows the use of office visits. After a steep drop early in the pandemic (March–May 2020), from June 2020 onward the average monthly claims were 10% below pre-COVID trends (*p* < 0.001). The second panel shows that telehealth visits had a significant spike immediately after COVID and remained high during the ongoing COVID pandemic (an average of 14,700 claims per 100,000 beneficiaries per month from June 2020 through December 2022). Combining office and telehealth visits, shown in the third panel, total visits increased from June 2020–December 2022 by 9% relative to the predicted rate (*p* < 0.001).

 The fourth panel shows the use of acute care: Inpatient stays or emergency department visits fell particularly rapidly from March–May 2020 (-35%; *p* < 0.001) and remained low after that (-9% from June 2020; *p* < 0.001) relative to prediction, until converging back to pre-COVID levels in spring 2022.

The fifth panel shows residence in long-term care facilities, excluding Medicare-paid skilled nursing facilities. From March to May 2020, average monthly claims for long-term care were 17% below expected (*p* < 0.001) and remained 14% below expected through 2022 (*p* < 0.001).

The last panel shows monthly Medicare spending for the ADRD cohort. Throughout most of the period, per-capita Medicare spending remained relatively close to the predicted trend at roughly $2,568 per month or nearly $30,800 per year. Mean spending from June 2020 to December 2022 is 4 percent above the predicted value (*p* < 0.001). The near-constancy of spending in the ADRD cohort differs from the general population, where Medicare spending fell by 5% in 2020 [28]. In the ADRD cohort, there was a significant spike in Medicare spending at the end of 2020 (driven by the increase in COVID-19 cases due to the Delta variant of SARS-CoV-2). Spending remained on trend in 2021, but actual spending was above predicted in 2022, driven mainly by above-trend spending in outpatient and SNF services. Components of spending by service category are in Appendix Figure 2.

**Fig. 2:**
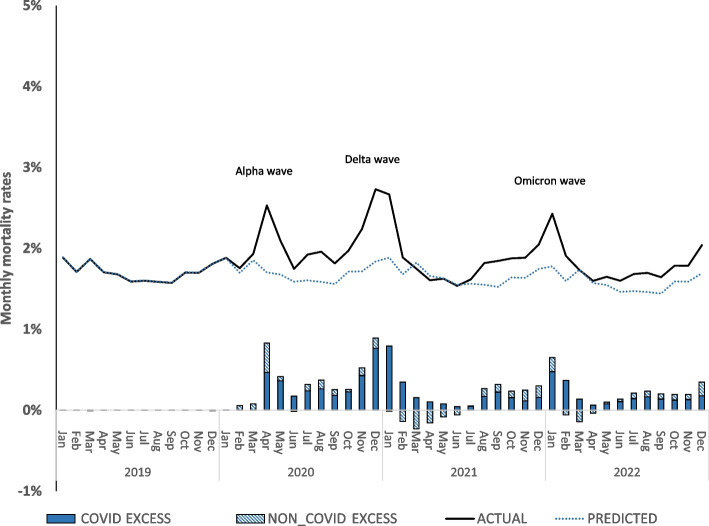
Monthly trends in actual and predicted mortality rates for patients with ADRD during the COVID-19 pandemic, March 2020-December 2022. Notes: COVID EXCESS: Excess deaths attributed to COVID if there was a COVID diagnosis in an inpatient, skilled nursing facility, or hospice claim in the last 14 days of life. NON-COVID EXCESS: Excess deaths not attributed to COVID (EXCESS minus COVID EXCESS). Data are for patients with ADRD who are age 66 or older and enrolled that month in fee-for-service Medicare Parts A and B, in administrative data from the Centers for Medicare and Medicaid Services (CMS) for a 100% sample of beneficiaries in traditional Medicare. Observations are at the patient-month level: *N* = 114,638,189 person-month observations; 5,237,349 unique beneficiaries. Predicted deaths are from OLS regression models using monthly data from January–December 2019, controlling for 10-year age-gender groups, race/ethnicity (non-Hispanic Black, Hispanic, Asian/Pacific Islander, other), dual Medicare-Medicaid enrollment, Social Deprivation Index (SDI) score tercile, urban/rural/isolated areas, 24 chronic conditions, month of the year, hospital referral region (HRR), and residence in a skilled nursing facility (SNF) for rehabilitative care in the prior month. Appendix Figure 1b shows results are the same when predicted from a logistic regression model

Estimates of COVID and non-COVID excess mortality are shown in Fig. 2. From June 2020 to December 2022, after the initial wave of COVID deaths, there were 231,000 excess deaths, 15.4% above the predicted value (*p* < 0.001). Most of the above-predicted rates are driven by three major variants of SARS-CoV-2: Alpha, Delta, and Omicron, with the Delta wave having the most significant impact on ADRD excess deaths. Over the entire June 2020-December 2022 period, 89% of excess deaths are estimated to be due to COVID-19. In the second half of 2021, there were more non-COVID deaths. For example, between August and December 2021, 60% of excess deaths are due to COVID, and 40% are due to non-COVID causes. Between January 2022 (when the Omicron wave peaked) and December 2022, 81% of excess deaths are estimated to be due to COVID-19.

### Racial and sociodemographic disparities in mortality

**Fig. 3:**
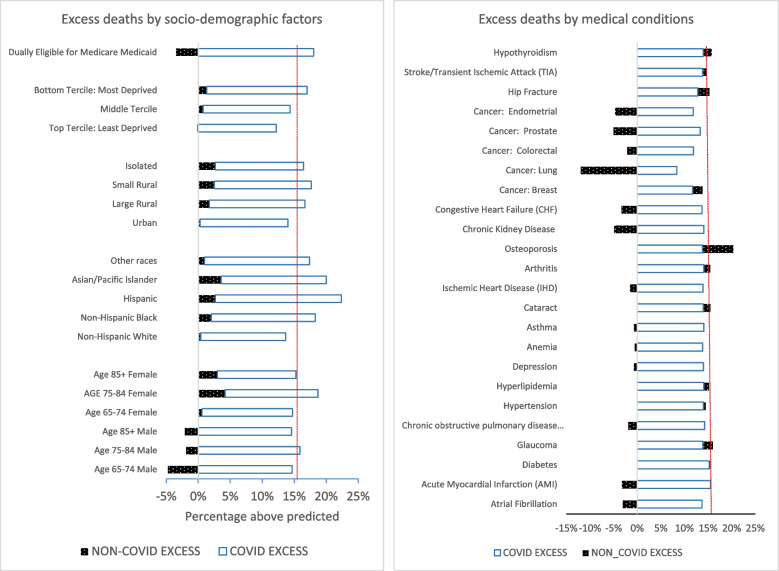
Excess deaths by sociodemographic factors and medical conditions for patients with ADRD during the COVID-19 pandemic, June 2020-December 2022. Notes: Dual eligibility for Medicare and Medicaid is used as a proxy for being a low-income beneficiary. Social Deprivation Index (SDI) scores are assigned by the Centers for Medicare and Medicaid Services (CMS) based on ZIP code by linking to 2014-2018 American Community Survey data. The SDI score ranges from 0-100, with a higher SDI score associated with the lower socioeconomic status of the zip code area. We group the SDI into terciles to allow for non-linear effects on utilization and outcomes. Federal Rural-Urban Commuting Area (RUCA) codes from 2010 are assigned by CMS based on census tract. Race/ethnicity are categorized using the Research Triangle Institute race and ethnicity indicator, which identifies more beneficiaries as Hispanic based on surname COVID EXCESS: Excess deaths attributed to COVID if there was a COVID diagnosis in an inpatient, skilled nursing facility, or hospice claim in the last 14 days of life. NON-COVID EXCESS: Excess deaths not attributed to COVID (EXCESS minus COVID EXCESS). Data are for patients with ADRD who are age 66 or older and enrolled that month in fee-for-service Medicare Parts A and B, in administrative data from CMS for a 100% sample of beneficiaries in traditional Medicare. Observations are at the patient-month level: N = 114,638,189 person-month observations; 5,237,349 unique beneficiaries. Predicted deaths are from OLS regression models using monthly data from January–December 2019, controlling for 10-year age-gender groups, race/ethnicity (non-Hispanic Black, Hispanic, Asian/Pacific Islander, other), dual Medicare-Medicaid enrollment, Social Deprivation Index (SDI) score tercile, urban/rural/isolated areas, 24 chronic conditions, month of the year, hospital referral region (HRR), and residence in a skilled nursing facility (SNF) for rehabilitative care in the prior month. Chronic Conditions are from the Centers for Medicare and Medicaid Services (CMS) Data Warehouse (CCW)

Figure 3 shows the excess death rate for June 2020-December 2022 by race/ethnicity, age/gender, urban/rural location, and sociodemographic factors (dual Medicare/Medicaid eligibility and SDI). Starting from the bottom of the figure, overall excess deaths are slightly higher for females than males across different age groups (16.5% for females vs. 12.4% for males, *p* < 0.001). Non-COVID excess deaths are also higher for females across all age groups (average of 3% vs. -3% for men). Black and Hispanic patients had significantly higher excess deaths (each 18%) compared to White patients (14%; *p* < 0.001). Non-COVID excess deaths were also significantly higher in these groups. Excess mortality was 0.4% for non-Hispanic Whites compared to 2.1% for non-Hispanic Blacks (*p* < 0.001), 2.7% for Hispanics (*p* < 0.001), 3.6% for Asian/Pacific Islanders (*p* < 0.001); and 6.1% for people of other races (*p* < 0.001). Both COVID and non-COVID excess deaths were higher in rural and isolated zip codes as compared to urban areas (17%, 18%, and 16% in largely rural, small rural, and isolated zip codes as compared to 14% in the urban zip codes; *p*-value for each comparison vs. urban < 0.001). Excess deaths were also significantly higher for those in the bottom tercile of the social deprivation index (most deprived) than those in the top tercile/least deprived (17% vs. 12%; *p* < 0.001).

The second panel of Fig. 3 shows the excess deaths of people with various chronic conditions. There is no clear evidence of disparities for people with particular chronic conditions, except that excess deaths for people with cancer were lower, and excess mortality for people with osteoporosis was above average.

### Geographic variation across states

**Fig. 4:**
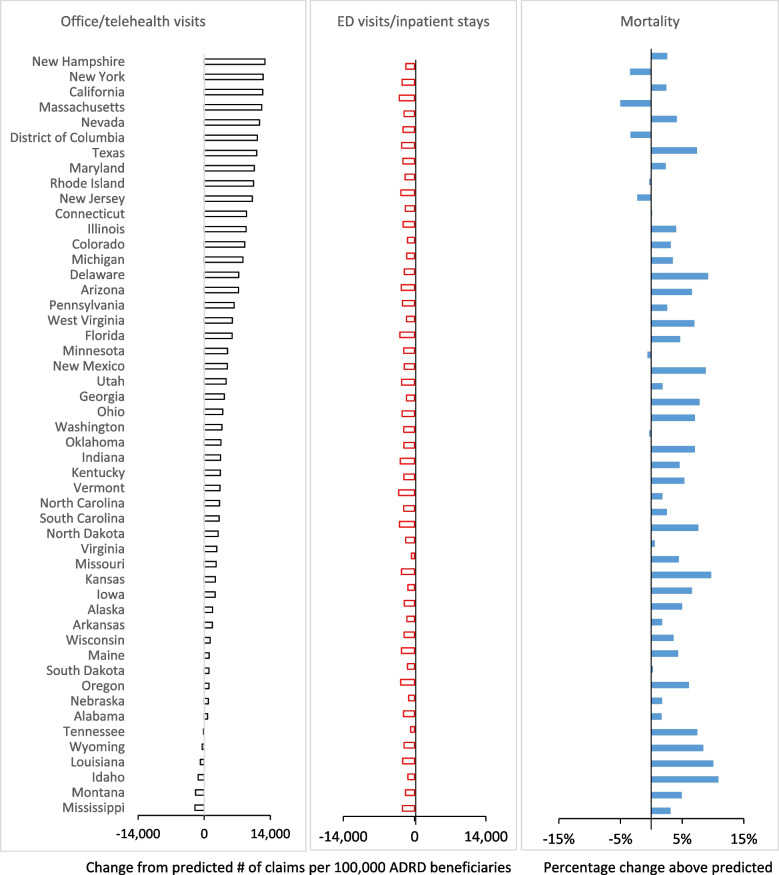
Geographic variation in actual versus predicted routine and acute care utilization and in mortality for patients with ADRD during COVID-19 pandemic, June 2020-December 2022. Note: Data are for patients with ADRD who are age 66 or older and enrolled that month in fee-for-service Medicare Parts A and B, in administrative data from the Centers for Medicare and Medicaid Services (CMS) for a 100% sample of beneficiaries in traditional Medicare. Observations are at the patient-month level: *N* = 114,638,189 person-month observations; 5,237,349 unique beneficiaries. Predicted utilization and deaths are from OLS regression models controlling for 10-year age-gender groups, race/ethnicity (non-Hispanic Black, Hispanic, Asian/Pacific Islander, other), dual Medicare-Medicaid enrollment, Social Deprivation Index (SDI) score tercile, urban/rural/isolated areas, 24 chronic conditions, month of the year, hospital referral region (HRR), and residence in a skilled nursing facility (SNF) for rehabilitative care in the prior month

Variation across states in medical care use and mortality for ADRD patients from June 2020 to December 2022 is shown in Fig. 4. While almost all states demonstrated a net increase in overall visits, the increased level varied significantly across states. In general, states in the northeast and mid-Atlantic regions had larger increases in visits compared to Southern states. The change in inpatient visits is shown in the second panel of Fig. 4, and mortality is in the third panel, each sorted by the change in office or telehealth visits.

**Fig. 5:**
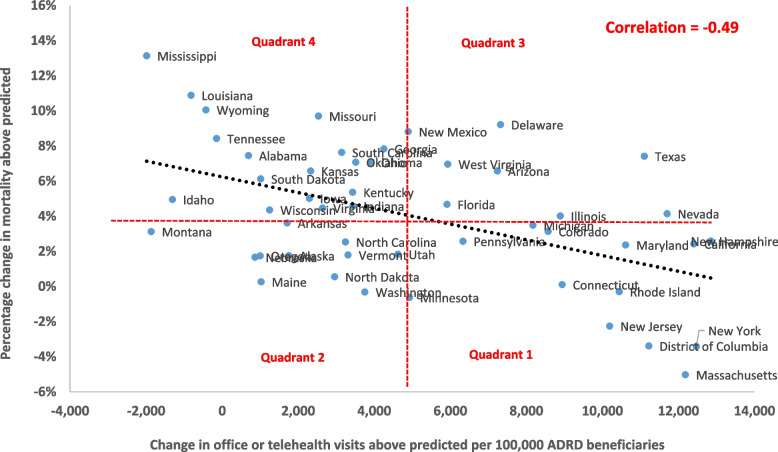
Change in actual versus predicted office or telehealth related to excess mortality for patients with ADRD during the COVID-19 pandemic, June 2020-December 2022. Note: Data are for patients with ADRD who are age 66 or older and enrolled that month in fee-for-service Medicare Parts A and B, in administrative data from the Centers for Medicare and Medicaid Services (CMS) for a 100% sample of beneficiaries in traditional Medicare. Observations are at the patient-month level: *N* = 114,638,189 person-month observations; 5,237,349 unique beneficiaries. Predicted utilization and deaths are from OLS regression models controlling for 10-year age-gender groups, race/ethnicity (non-Hispanic Black, Hispanic, Asian/Pacific Islander, other), dual Medicare-Medicaid enrollment, Social Deprivation Index (SDI) score tercile, urban/rural/isolated areas, 24 chronic conditions, month of the year, hospital referral region (HRR), and residence in a skilled nursing facility (SNF) for rehabilitative care in the prior month

States with larger increases in office or telehealth visits during the pandemic had the lowest excess deaths, with a significant negative overall correlation coefficient of -0.49 across states. Figure 5 depicts the relationship between changes in mortality and the combination of office and telehealth visits. The vertical and horizontal red lines show the averages across all states. Quadrant 1 shows states with above-average increases in office or telehealth visits and below-average excess mortality. The states with the lowest excess mortality in this quadrant are Massachusetts and New York. Quadrant 4 shows states with excess mortality significantly above average, and office and telehealth visits below average. The two states in this quadrant with the highest excess mortality are Mississippi and Louisiana. Appendix Figure 3 shows a weaker relationship between office or telehealth and inpatient/ED visits, *r* = -0.24.

## Discussion

The COVID pandemic was particularly hazardous for those living in group settings and with pre-existing chronic disease, which includes the ADRD population. This paper aimed to determine how much COVID-19 affected the medical care receipt and health outcomes of ADRD patients. Because ADRD patients are sicker than average, one might expect COVID-19 to have had a large negative effect on this group. On the other hand, the switch from in-person to telehealth visits made care much more accessible for people with ADRD, potentially improving outcomes.

Our analysis yields several results. First, we found a net increase in medical care receipt for ADRD patients during the COVID era compared to the pre-COVID era. In-person medical care visits declined, but an increase in telehealth visits more than compensated for the decline in office visits. At the same time, there was a significant reduction in care received in an emergency setting, even accounting for people needing care for COVID-19. Medicare per capita spending aligned with predicted rates during 2020 and 2021, with spending above the trend in 2022, driven mainly by outpatient and SNF services. This may reflect the recovery of these services in 2022 after declines in 2020 and 2021.

Living arrangements for people with ADRD changed markedly with COVID. Long-term residential care plummeted at the pandemic’s start and never fully recovered. Even by December 2022, the use of care in long-term residential facilities was 10 percent lower than expected based on pre-COVID levels.

The COVID-19 pandemic was associated with high excess mortality among older adults with ADRD. From June 2020 to December 2022, there were 231,000 excess deaths in this population, 16% above the prediction. Most of these deaths were attributable to COVID, though a share were attributable to non-COVID causes.

It is difficult to know how much the uptake of telemedicine affected health trends for people with ADRD. States with greater increases in office and telehealth visits had smaller increases in excess deaths and greater declines in inpatient/ED visits, which may reflect that these visits prevented adverse events. While this does not establish a causal connection, it is consistent with such a hypothesis. However the percentage of excess deaths attributable to causes other than COVID-19 may also be an important metric. COVID was the primary cause of the bulk of the deaths from 2020–2022, suggesting that deterioration of non-COVID care was not a significant issue for this population. However, these trends were slightly different at different times, especially in the second half of 2021, when non-COVID excess deaths accounted for 40% of excess deaths. This may reflect a decline in the appropriate use or quality of care during that time.

In addition to geographic disparities, substantial sociodemographic disparities persist in utilization and excess deaths among ADRD patients. Death rates increased more among ADRD patients who were Black, other race, or Hispanic. It also increased most among those living in the lowest SES zip codes and isolated rural areas. This is consistent with other data [28] showing that COVID-19 exacerbated existing racial and socioeconomic disparities among traditional Medicare beneficiaries, both in terms of deaths due to COVID-19 and those due to non-COVID factors.

### Limitations

Our study has several limitations. First, we only include ADRD beneficiaries enrolled in traditional Medicare, omitting those in Medicare Advantage. Also, we focus only on Medicare beneficiaries over age 65. However, this did not likely affect our results as most ADRD patients are in this age group and are in traditional Medicare [29]. In addition, we use administrative data to determine race and ethnicity, which are less accurate than self-reports for Hispanics [24]. Our assignment of diagnoses based on codes in claims data may miss some people who have conditions but did not have codes for those conditions in their medical claims. Finally our measure of COVID mortality was based on a COVID claim in the 2 weeks prior to death and not death certificate recording. This captured those who sought medical care for their symptoms and received a COVID diagnosis. Testing capacity increased over the course of the pandemic, especially in the first several months. Thus, our results will be more accurate in terms of deaths attributable to COVID in 2021 and 2022.

## Conclusions

Older adults with ADRD had substantial deaths above pre-pandemic projections during the COVID-19 pandemic. Most of these were attributed to COVID-19, but a significant minority were non-COVID deaths. A dramatic increase in telehealth visits that was uneven across states was strongly associated with lower excess deaths from both COVID-19 and non-COVID causes.

### Supplementary Information

Supplementary Material 1.

## Data Availability

The data that support the findings of this study are available from The Centers for Medicare and Medicaid Services (CMS), but were obtained through a data use agreement for the current study, and so are not publicly available.

## Abbreviations

ADRD: Alzheimer’s Disease and Related Dementias
COVID: Coronavirus disease 2019
SARS-CoV-2: Severe acute respiratory syndrome coronavirus 2
CCW: Chronic Conditions Data Warehouse
E&M: Evaluation and management visits
ED: Emergency department utilization
HRR: Hospital referral region
LTC: Long-term care services
SNF: Skilled nursing facility
SDI: Social Deprivation Index
