# Supplementary material for: Medical visits and mortality among dementia patients during the COVID-19 pandemic compared to rates predicted from 2019
Source: BMC Geriatr. 2024 Sep 2;24:727. doi: 10.1186/s12877-024-05298-2 (PMC11367830; doi:10.1186/s12877-024-05298-2)
Supplement: Supplementary file 1 — Supplementary Material 1. [file 12877_2024_5298_MOESM1_ESM.docx]

APPENDIX

to Ghosh et al., **Medical visits and mortality among dementia patients during the COVID-19 pandemic compared to rates predicted from 2019**

**Appendix Table 1: Codes to identify Alzheimer's Disease and Related Dementia (ADRD)**

| **CCW label** | Alzheimer's Disease and Related Disorders or Senile Dementia (ADRD) |  |
| --- | --- | --- |
| **Number/Type of Claims to Qualify:** | At least 1 inpatient, SNF, HHA, HOP, or Carrier claim with DX code |  |
| **Valid ICD-10 Codes** | F01.50, F01.51, F02.80, F02.81, F03.90, F03.91, F04, F05, F06.1, F06.8, G13.8, G30.0, G30.1, G30.8, G30.9, G31.01, G31.09, G31.1, G31.2, G94, R41.81, R54 (any DX on the claim) |  |
|  |  |  |
|  |  |  |
|  |  |  |
|  |  |  |

| **Appendix Table 2: Codes used to identify service utilization and spending, death, and COVID-19 diagnosis** | | |  |
| --- | --- | --- | --- |
| **Visit** | **Type** | **Code** | |
| Office visits | CPT/HCPCS | Office visits: 99202-99215 Wellness visits: G0402, G0438, G0439 | |
| Telemedicine | CPT/HCPCS | Physician telephone Services: 99441–99443 Non-physician Telephone Services: 98966–98968 Interprofessional internet consultations: 99446–99452 Remote Patient Monitoring: 99453–99454  Eval. of video/images: G2010, Brief communication technology-based service, e.g., virtual check-in: G2012 Online digital evaluation/management: 99421–99423 Online assessment and management: G2061–G2063 | |
|  |  |  |  |
|  |  | Initial telehealth consultations: G0425–G0427 Inpatient telehealth pharmacologic management: G0459 Follow-up inpatient consultation G0406–G0408 Video-conferenced Critical Care: 0188T Critical care consultation: G0508–G0509 Virtual visit, rural or shortage area: G0071 Physiologic Monitoring 99457 Distant site service, rural or shortage area: G2025, Continous glucose monitoring, subcutaneous sensor: 95250–99251(analysis, interpretation and report) Collection and interpretation of physiologic data: 99091 | |
|  |  |  |  |
|  | hcpcs_1ST_MDFR_CD  hcpcs_2ND_MDFR_CD | Interactive audio/video telecommunication system: GT Asynchronous telecommunications system: GQ Synchronous conference between with provider: 95 Diagnosis/eval./treatment acute stroke symptoms: G0 | |
|  | LINE_PLACE_OF_SRVC_CD | Unassigned, N/A: 02 | |
| Urgent/Emergent admission | CLM_IP_ADMSN_TYPE_CD | 1 (Emergency), 2 (Urgent) | |
| LTC | CPT/HCPCS  Place of service code | Nursing facility eval./management visit: 99304–99310 Nursing facility discharge management: 99315–99316 Annual nursing facility assessment: 99318  Third-party mandated:32, Preventive services: 33 | |
| ED visits | REV_CNTR_CODE | Emergency room: 0450–0459, ER professional fee:0981 | |
| Medicare Spending | Medicare + Out-of-pocket | Inpatient, Outpatient, Carrier, SNF, Hospice, Home Health, Durable Medical Equipment (DME) | |
| Deaths | Date of Death | Date of Death | |
| COVID-19 | ICD10- Diagnosis code | COVID-19: U0171, Assoc. pneumonia: J1281/ J1282 | |

Appendix Table 3 continued

**Appendix Figure 1a: Monthly trends in actual and predicted office and telehealth visits for patients with ADRD during the COVID-19 pandemic, March 2020-December 2022**

**Appendix Figure 1b: Monthly trends in mortality rates for patients with ADRD during the COVID-19 pandemic: actual and predicted using OLS and logistic regression**

**Notes, Figures 1a and 1b:** Dashed lines are predictions from regression models using monthly data from January–December 2019. Data are for patients with ADRD who are age 66 or older and enrolled that month in fee-for-service Medicare Parts A and B, in administrative data from the Centers for Medicare and Medicaid Services (CMS) for a 100% sample of beneficiaries in traditional Medicare. Observations are at the patient-month level: N = 114,638,189 person-month observations; 5,237,349 unique beneficiaries. Predicted utilization and mortality is from OLS regression models using monthly data from January–December 2019, controlling for 10-year age-gender groups, race/ethnicity (non-Hispanic Black, Hispanic, Asian/Pacific Islander, other), dual Medicare-Medicaid enrollment, Social Deprivation Index (SDI) score tercile, urban/rural/isolated areas, 24 chronic conditions, month of the year, hospital referral region (HRR), and residence in a skilled nursing facility (SNF) for rehabilitative care in the prior month (except in the long-term care facility regression analysis).

**Appendix Figure 2: Monthly trends in actual and predicted spending by service category for patients with ADRD during the COVID-19 pandemic, March 2020-December 2022**

Note: Data are for patients with ADRD who are age 66 or older and enrolled that month in fee-for-service Medicare Parts A and B, in administrative data from the Centers for Medicare and Medicaid Services (CMS) for a 100% sample of beneficiaries in traditional Medicare. Observations are at the patient-month level: N = 114,638,189 person-month observations; 5,237,349 unique beneficiaries. Predicted spending is from OLS regression models controlling for 10-year age-gender groups, race/ethnicity (non-Hispanic Black, Hispanic, Asian/Pacific Islander, other), dual Medicare-Medicaid enrollment, Social Deprivation Index (SDI) score tercile, urban/rural/isolated areas, 24 chronic conditions, month of the year, hospital referral region (HRR), and residence in a skilled nursing facility (SNF) for rehabilitative care in the prior month.

Appendix Figure 3: Change in actual versus predicted office or telehealth related to change in inpatient or ED visits for patients with ADRD during the COVID-19 pandemic, June 2020-December 2022

Note: Data are for patients with ADRD who are age 66 or older and enrolled that month in fee-for-service Medicare Parts A and B, in administrative data from the Centers for Medicare and Medicaid Services (CMS) for a 100% sample of beneficiaries in traditional Medicare. Observations are at the patient-month level: N = 114,638,189 person-month observations; 5,237,349 unique beneficiaries. Predicted utilization and deaths are from OLS regression models controlling for 10-year age-gender groups, race/ethnicity (non-Hispanic Black, Hispanic, Asian/Pacific Islander, other), dual Medicare-Medicaid enrollment, Social Deprivation Index (SDI) score tercile, urban/rural/isolated areas, 24 chronic conditions, month of the year, hospital referral region (HRR), and residence in a skilled nursing facility (SNF) for rehabilitative care in the prior month.
